# Optoelectrically controlled transistors in graphene-based valley-gapless, indirect-gap, and spin-valley-gapless semiconductors

**DOI:** 10.1016/j.isci.2026.116289

**Published:** 2026-06-05

**Authors:** Xiao-Long Lü, Ze-Han Hu, Xin-Zhi Liu, Yan-Chao Zhang, Jia-En Yang

**Affiliations:** 1 College of Science, Guangxi University of Science and Technology, Liuzhou, Guangxi 545006, China; 2 College of Physics, Chongqing University, Chongqing 401331, China; 3 School of Electronics and IoT, Chongqing Polytechnic University of Electronic Technology, Chongqing 401331, China

**Keywords:** Condensed matter physics, Electrical engineering, Nanomaterials

## Abstract

Generating and modulating fully spin-, valley-, and spin-valley-polarized currents are key to spintronics, valleytronics, and spin-valleytronics, respectively. Here, we propose optically controlled graphene-based multi-gapless semiconductors (SCs) for electrical modulation of such currents. The results reveal that antiferromagnetic exchange fields and a modified Haldane model induce valley-gapless SCs, which transition into four distinct spin-valley-gapless SCs under off-resonant circularly polarized light. Moreover, these gapless SCs, with the spin and valley degrees of freedom tunable via a gate voltage, are switched to indirect-gap SCs as the optical intensity increases. Consequently, under a gate voltage, optically modulated valley-gapless SCs, spin-valley-gapless SCs, and indirect-gap SCs, respectively give rise to fully spin-polarized currents, spin-valley-polarized currents, and an “off” state, thus enabling the realization of optoelectrically controlled transistors. Additionally, electrostatic potentials on leads further regulate current magnitude. Our work highlights the importance of these gapless SCs for optoelectrically controlled valleytronic and spin-valleytronic devices.

## Highlights

• Optically controlled graphene multigapless semiconductors enable spin-valley polarization
• Gate voltage switches between fully spin- and spin-valley-polarized currents
• Electrostatic potentials can regulate current magnitude
• A concept of spin-valley-gapless semiconductors is introduced

## Graphical abstract

**Figure:**
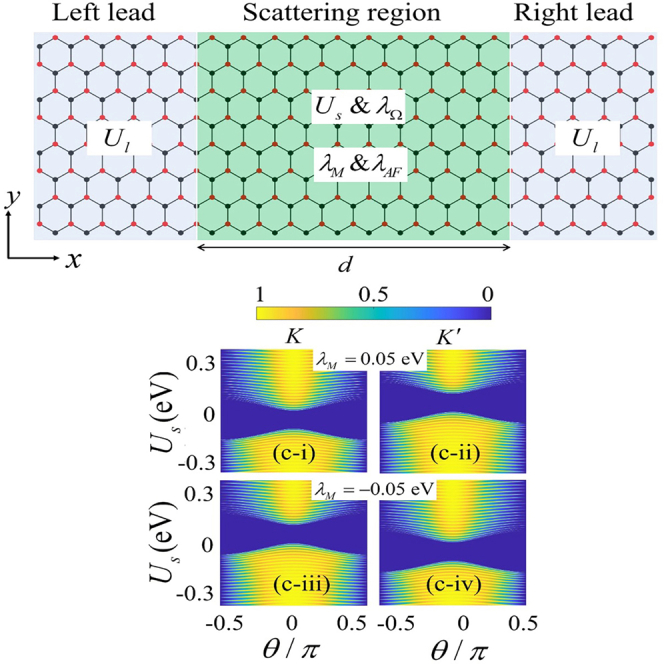

## Introduction

Graphene^1^ has triggered numerous studies to investigate topological effects^2^^,^^3^^,^^4^^,^^5^^,^^6^ and electronic transport,^7^^,^^8^^,^^9^^,^^10^^,^^11^ driven by its fascinating physical properties and promising prospects for device design. Motivated by graphene, silicene,^12^ germanene,^13^ stanene,^14^ and transition metal dichalcogenides^15^ garnered extensive theoretical and experimental investigation, which can serve as a platform for spintronics,^16^ valleytronics,^17^ and spin-valleytronics,^18^ analogous to graphene. The two inequivalent valleys *K*′ and *K* of graphene, which are separated well in reciprocal space,^19^^,^^20^^,^^21^ suppress the intervalley scattering, establishing the valley index as an independent intrinsic degree of freedom in addition to charge and spin. As a result, the valley index can be utilized to generate valley-polarized currents for valleytronics capable of storing and processing digital information. It also facilitates spin-valley coupling for spin-valleytronics. Moreover, the tunable bulk band gaps of these two valleys in these materials enable the emergence of abundant spin- and valley-related transport phenomena,^22^^,^^23^^,^^24^^,^^25^^,^^26^ warranting investigation into their transport mechanisms and related applications in devices.

The fundamental challenge for designing valleytronics and spin-valleytronics lies in generating and manipulating valley- and spin-valley-polarized currents. Recently, modulated approaches in two-dimensional materials with a honeycomb lattice structure have been proposed to enable these polarization currents, which can be attributed to breaking spin and valley degeneracies and inducing band offsets^27^^,^^28^ between different components in the junction. For instance, strain,^29^^,^^30^^,^^31^ as a crucial method, can be utilized to modulate the spin and valley degrees of freedom, by introducing a pseudovector potential. In the presence of Rashba effects,^32^^,^^33^^,^^34^ abundant valley and spin-valley-polarized edge states emerge, facilitating robust devices. Compared to the monolayer systems above, the bilayer systems^35^^,^^36^^,^^37^ also provide an important platform to enable the emergence of tunable polarization currents. In addition, it is worth mentioning that off-resonant circularly polarized light (CPL)^9^^,^^22^^,^^38^^,^^39^^,^^40^ in some modulated methods has received much attention owing to its exceptional controllability and rich physical properties.

Inspired by the spin-gapless semiconductor (SC),^41^^,^^42^^,^^43^ which enables electrically tunable spin-polarized current, the valley-gapless SC^10^^,^^44^ offers an alternative route to generate valley-polarized current. These SCs feature gapless bands at the Fermi energy, hosting fully spin- and valley-polarized electron-hole pairs. Similar to two types of valley-gapless SC,^10^ the rarely reported concept of spin-valley-gapless SC can be naturally proposed to induce corresponding polarization currents with electrical manipulation. The spin-valley-gapless SCs we focus on are characterized by the touching between the conduction band edge of one valley coupled with one spin and the valence band edge of the other valley coupled with opposite spin at the Fermi energy. As a result, the electron carriers contribute to one type of fully spin-valley-polarized current, while the hole carriers produce another, yielding four distinct types.

In this work, we introduce the concept of the spin-valley-gapless SCs, along with its generation, phase transitions, and electrically tunable transport. Within a modified Haldane model incorporating antiferromagnetic (AFM) exchange field, two valley-gapless SCs emerge in graphene. When the off-resonant CPL is further incorporated, four distinct spin-valley-gapless SCs arise from the valley-gapless SC, which can be controlled by the optical intensity and polarization direction. Furthermore, these gapless SCs can transition to the indirect-gap SCs as the optical intensity increases, thereby suppressing carrier propagation. Based on the transport signatures of the gapless and indirect-gap SCs, a graphene-based two-terminal device, where the optically controlled SCs are applied to the scattering region and the leads host the metallic phase, can enable the “on” state with fully valley- and spin-valley-polarized currents, and the “off” state, under a gate voltage in the scattering region, thereby realizing an optoelectronic-modulated transistor. These findings highlight the promising potential of spin-valley gapless SCs for valleytronics and spintronics applications.

### Model and methods

We propose an optoelectronic-modulated transistor based on the valley-gapless SC, spin-valley-gapless SC, and indirect-gap SC. The monolayer graphene-based junction is shown schematically in Figure 2A, where *U*_*l*_ is the electrostatic potential in the two leads with a metallic phase, while a gate voltage *U*_*s*_, an off-resonant CPL and an AFM exchange field are applied to the scattering region with a modified Haldane model. Detailed computational methods for the proposed junction are provided in the STAR Methods.

## Results

### Band structures of valley-gapless, spin-valley-gapless, and indirect-gap SCs

**Figure 1:**
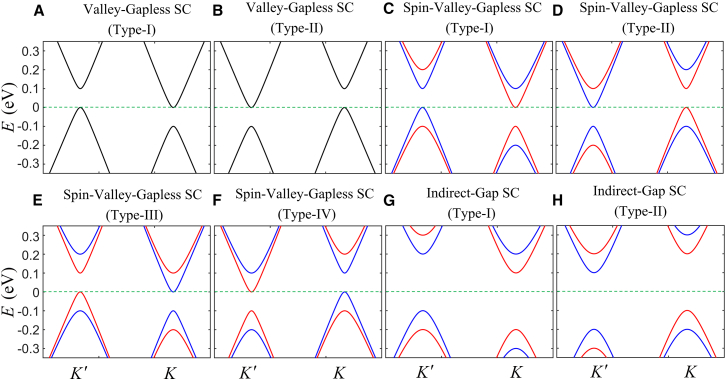
Band structures of valley-gapless, spin-valley-gapless, and indirect-gap SCs (A) *λ*_Ω_ = 0, *λ*_*M*_ = 0.05 eV, *λ*_*AF*_ = 0.05 eV. (B) *λ*_Ω_ = 0, *λ*_*M*_ = −0.05 eV, *λ*_*AF*_ = 0.05 eV. (C) *λ*_Ω_ = 0.1 eV, *λ*_*M*_ = 0.05 eV, *λ*_*AF*_ = 0.05 eV. (D) *λ*_Ω_ = −0.1 eV, *λ*_*M*_ = 0.05 eV, *λ*_*AF*_ = 0.05 eV. (E) *λ*_Ω_ = 0.1 eV, *λ*_*M*_ = −0.05 eV, *λ*_*AF*_ = 0.05 eV. (F) *λ*_Ω_ = −0.1 eV, *λ*_*M*_ = −0.05 eV, *λ*_*AF*_ = 0.05 eV. (G) *λ*_Ω_ = 0.2 eV, *λ*_*M*_ = 0.05 eV, *λ*_*AF*_ = 0.05 eV. (H) *λ*_Ω_ = 0.2 eV, *λ*_*M*_ = −0.05 eV, *λ*_*AF*_ = 0.05eV. The green dotted line depicts the Fermi energy. The black, red, and blue lines depict the spin-degenerate, spin-up, and spin-down modes, respectively.

**Figure 2:**
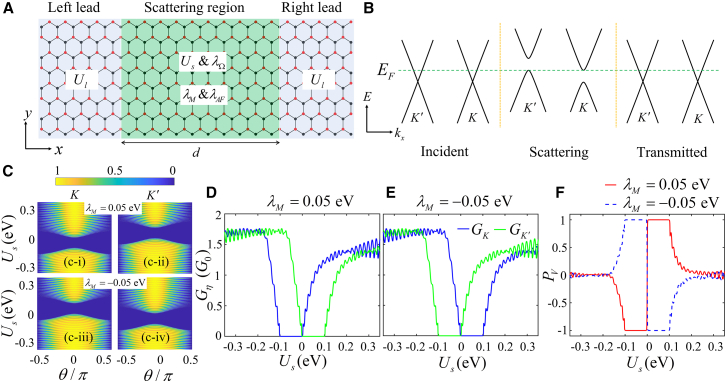
Gate-controlled valley-gapless SC-based valley filter (A) Schematic of the gate-controlled valley-gapless SC-based valley filter where the gate voltages *U*_*s*_,*U*_*l*_ are applied to the scattering region with the length *d*, and the two leads, respectively. (B) The mechanism for the valley filter effect in (A) is that for a positive (negative) voltage *U*_*s*_, the electron incident from the left lead with the Fermi energy *E*_*F*_ = 0 at the valley *K*′(*K*) can be transmitted into the right lead. (C) The valley-dependent transmission probability in the (*U*_*s*_,*θ*) plane, where *θ* is the incident direction of the electron. The left (right) images depict the valley-dependent transmission probability at the valley *K*(*K*′), and the upper and lower images correspond to the values of *λ*_*M*_ = ±0.05 eV. (D and E) The gate-controlled conductances as a function of *U*_*s*_ for two values of *λ*_*M*_ = ±0.05 eV. (F) The valley polarization efficiencies are presented for (D) and (E). The fixed parameters are set to *λ*_Ω_ = 0, *λ*_*AF*_ = 0.05 eV, *U*_*l*_ = −0.1 eV, and *d* = 450*a* with the lattice constant *a* = 0.426 nm.

The metallic phase of graphene can undergo transitions to the valley-gapless SCs, spin-valley-gapless SCs, and indirect-gap SCs, respectively under the modified Haldane model, AFM exchange field, and off-resonant CPL. Figures 1A and 1B demonstrate that with the fixed AFM exchange field, two types of valley-gapless SCs can be generated and tuned by the modified Haldane model. More interestingly, when the off-resonant CPL is further introduced, two types of valley-gapless SCs are transformed into four types of spin-valley-gapless SCs, as shown in Figures 2C–2F) and controlled by the polarization direction and the modified Haldane model. As the intensity of off-resonant CPL increases, these gapless SCs are converted into the indirect-gap SCs as shown in Figures 1G and 1H, which can be utilized to cut off the current. The mechanism of the formation of these SCs stems from Equation 2, where spin- and valley-dependent eigenvalues determine the band shift.

### Gate-controlled valley-gapless SC-based valley filter

Furthermore, the valley-gapless SCs can give rise to the valley-filter effect due to their transport properties in a two-terminal device in Figure 2A, where the electrostatic potential *U*_*l*_ (gate voltage *U*_*s*_) is applied to the two leads (scattering region). In order to demonstrate the valley-filter effect, the corresponding mechanism is presented in Figure 2B, where the Fermi energy *E*_*F*_ = 0 crosses the bottom and top of the valence and conduction bands in the scattering region with *U*_*s*_ = 0. It demonstrates that the propagation of carriers is prohibited from passing through the scattering region for *U*_*s*_ = 0, wherein carriers coupled to valley degree of freedom can flow freely for *U*_*s*_≠0. When the two types of the valley-gapless SCs in Figures 1A and 1B are applied to the scattering region, the corresponding valley-dependent transmission probabilities in the (*U*_*s*_,*θ*) plane are shown in Figure 2C. These transmission probabilities exhibit that the valley degree of freedom can be tuned by the gate voltage, thus giving rise to the valley-resolved conductance, as shown in Figures 2D and 2E. Additionally, the valley-resolved conductance is fully valley-polarized, as confirmed by the valley polarization in Figure 2F, resulting in an electrically controlled valley-filter effect based on valley-gapless SCs.

**Figure 3:**
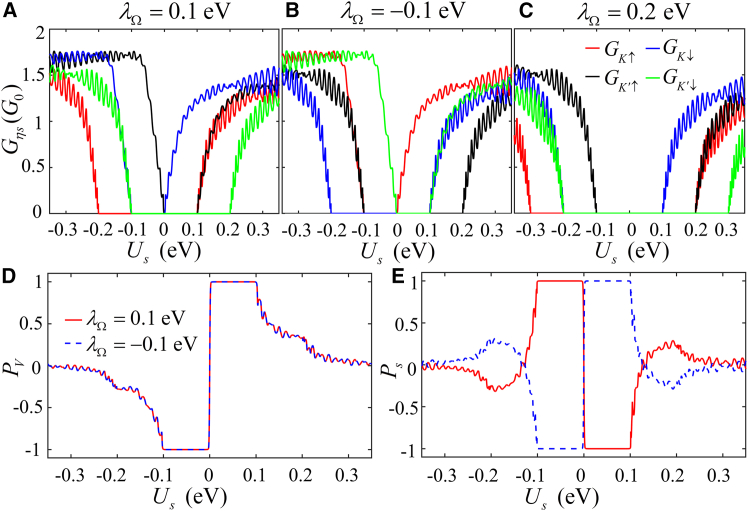
The gate-controlled spin-valley-dependent conductance and spin-valley polarization efficiency can be modulated by off-resonant CPL (A) *λ*_Ω_ = 0.1 eV. (B) *λ*_Ω_ = −0.1 eV. (C) *λ*_Ω_ = 0.2 eV. (D and E) The corresponding valley and spin polarization efficiencies are for (A) and (B), respectively. The fixed parameters are set to *λ*_*M*_ = 0.05 eV, *λ*_*AF*_ = 0.05 eV, *U*_*l*_ = −0.1 eV, and *d* = 450*a*. Note that the off-resonant CPL is applied solely within the scattering region, as depicted in Figure 2A.

Additionally, the optically controlled spin-valley-gapless and indirect-gap SCs can provide a platform for generating spin-valley-filter and gap effects, respectively, giving rise to the light-based transistor. When the off-resonant CPL is further considered, the fully valley-polarized conductance can be switched into the spin-valley-polarized one, as shown in Figure 3A, where only the spin-up or spin-down conductance with the valley *K*′ or *K* exists in the range of *U*_*s*_ ∈ [−0.1 eV, 0.1 eV]. It is worth noting that when the right polarization direction is converted into the left polarization direction, the other two spin-valley-dependent conductances are induced in the identical range of *U*_*s*_, as shown in Figure 3B. Moreover, the two cases in Figures 3A and 3B exhibit both the fully spin polarization and valley polarization, confirmed by the corresponding valley and spin polarization efficiencies in Figures 3D and 3E, respectively. The increasing intensity of CPL can transition spin-valley-gapless SCs into indirect-gap SC, providing an off state in the range of *U*_*s*_ ∈ [−0.1 eV, 0.1 eV] in Figure 3C. As a result, the transistor, accompanied by the on state with fully spin-valley-polarized conductance and the off state, can be modulated by the gate voltage and CPL.

### The effect of *U*_*l*_ on the spin-valley-dependent conductance

**Figure 4:**
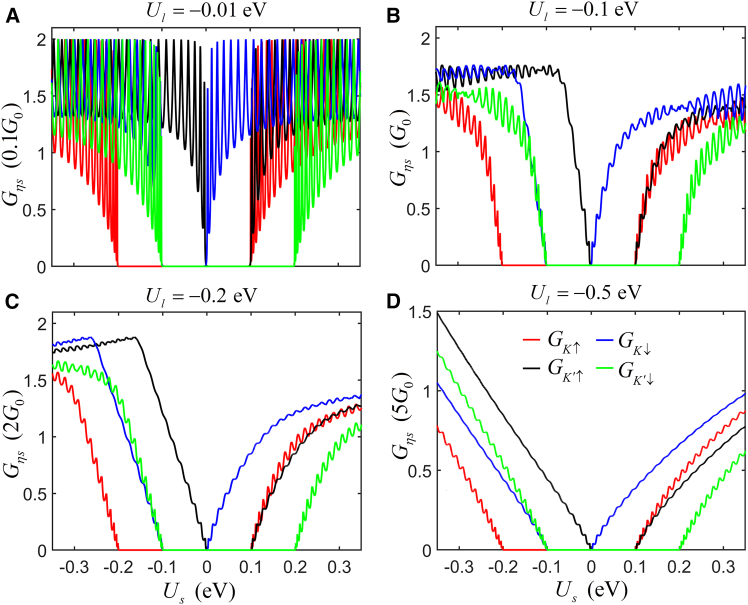
The effect of *U*_*l*_ on the spin-valley-dependent conductance (A) *U*_*l*_ = −0.01 eV for the conductance in the unit of 0.1*G*_0_. (B) *U*_*l*_ = −0.1 eV for the conductance in the unit of *G*_0_. (C) *U*_*l*_ = −0.2 eV for the conductance in the unit of 2*G*_0_. (D) *U*_*l*_ = −0.5 eV for the conductance in the unit of 5*G*_0_. The spin-valley-dependent conductance selected from Figure 3A is shown in (A–C), and (D).

Meanwhile, the electrostatic potential *U*_*l*_ applied to the two leads can increase conductance of valley and spin-valley-gapless SCs. The spin-valley-dependent conductance in Figure 3A is chosen to discuss the effect of *U*_*l*_. Compared to the original conductance in the unit of *G*_0_ with *U*_*l*_ = −0.1 eV in Figure 4B, the conductance in the unit of 0.1*G*_0_ for *U*_*l*_ = −0.01 eV in Figure 4A obviously becomes smaller. It can be observed that the conductance oscillates dramatically, which is attributed to the Fabry-Pérot interference.^32^ Specifically, the condition *mλ*_*F*_ = 2*L* forms the basis of Fabry-Pérot resonance, where *m* is a positive integer, *L* denotes the length of the scattering region, and *λ*_*F*_ is the de Broglie wavelength corresponding to the Fermi energy *E*_*F*_. Based on Equation 2, *U*_*s*_ at which the Fabry-Pérot resonances take place obeys ${\left[E-U_{s}-\eta \lambda_{M}\right]}^{2}=\hbar^{2}{v_{F}}^{2}m^{2}\pi^{2}/L^{2}+{\left(\eta \lambda_{\Omega }+s\lambda_{AF}\right)}^{2}$. This equation elucidates the physical origin of the conductance resonance phenomenon under the modulation of *U*_*s*_.

Additionally, when the absolute value of *U*_*l*_ increases, the corresponding conductance becomes larger in the units of 2*G*_0_ and 5*G*_0_, as shown in Figures 4C and 4D, respectively. Although *U*_*l*_ can enhance the tunneling, the conductance cannot keep increasing indefinitely. Based on Equations 4a and 4c, as the absolute value of *U*_*l*_ increases, the perpendicular wave vector *k*_*y*_ increases accordingly, resulting in the parallel wave vector $k_{x}^{II}$ in the scattering region gradually decreasing to the evanescent mode. As a result, the range of incident angles gradually decreases with an increase in the absolute value of *U*_*l*_, so the conductance cannot increase continuously.

Compared to prior work,^14^ this study proposes a novel concept of spin-valley gapless SC via band engineering, constructing a system of spin-valley-gapless SCs and their phase transition variants (valley-gapless, spin-valley-gapless, and indirect-gap SC). Its transport mechanism diverges fundamentally: using gate-voltage-controlled band mismatch to screen carriers for polarized currents, unlike prior reliance on electric-field modulation. The gate-voltage method offers simpler operation, higher precision, and direct valley-spin-valley band alignment adjustment for flexible current switching. Additionally, it enables active phase transition control via CPL and introduces potential-controlled current intensity via lead electrostatic potentials.

## Discussion

In summary, we have investigated the generation, modulation, and optoelectrically controlled transport properties of valley-gapless, spin-valley-gapless, and indirect-gap SCs in a two-terminal device. In the presence of an AFM exchange field, a modified Haldane model gives rise to two distinct types of valley-gapless SCs. Went off-resonant CPL is considered, two valley-gapless SCs evolve into four spin-valley-gapless SCs, which can be controlled by the optical polarization direction. Moreover, all gapless SCs can transition to the indirect-gap SCs as the optical intensity increases, thus realizing an “off” state. The valley-gapless SCs, in which the valley degree of freedom is electrically tunable via the gate voltage, can act as a platform to generate a fully valley-polarized current through electrical manipulation, realizing a valley-filter effect. Similar to valley-gapless SCs, spin-valley-gapless SCs also induce a fully spin-valley-polarized current with optical and electrical manipulation, giving rise to a spin-valley-filter effect. These fully polarized currents, controlled by a gate voltage, can be tuned off by increasing the optical intensity for both the valley and spin-valley valves. As a result, optoelectrically controlled transistors are formed. In addition, the electrostatic potential applied to the leads plays a crucial role in modulating the magnitude of the polarization currents. Our findings indicate that these SCs have very promising applications in spintronic and spin-valleytronic devices.

### Limitations of the study

This work proposes optically controlled graphene-based multi-gapless SCs to electrically generate fully spin-, valley-, and spin-valley-polarized currents via AFM exchange fields and a modified Haldane model. However, the study neglects the influence of low-frequency light, which may alter the band structures and spin-valley configurations beyond the scope of the current high-frequency approximation.

## Resource availability

### Lead contact

Further information and requests for resources should be directed to and will be fulfilled by the lead contact, Xiao-Long Lü (physicslxl@163.com).

### Materials availability

This study did not generate new unique material.

### Data and code availability

• All data reported in this paper will be shared by the lead contact upon request.
• This article does not report original code.
• Any additional information required to reanalyze the data reported in this article is available from the lead contact upon request.

## Acknowledgments

This work is supported by the National Natural Science Foundation of Guangxi Province (grant no. 2024GXNSFBA010229), Guangxi Science and Technology Base and Talent Project (grant no. Guike AD23026082), and 10.13039/501100001809National Natural Science Foundation of China (grant nos. 12304058 and 12365006).

## Author contributions

X.-L.L., writing – original draft, investigation, funding acquisition, and data curation; Z.-H.H. and X.-Z.L., software and visualization; Y.-C.Z. and J.-E.Y., software and visualization, writing – review and editing. All authors have read and agreed to the published version of the article.

## Declaration of interests

The authors declare no competing interests.

## STAR★Methods

### Key resources table

**Table:** 

REAGENT or RESOURCE	SOURCE	IDENTIFIER
**Software and algorithms**		

MATLAB	MathWorks	https://www.mathworks.com/products/matlab.html

### Experimental model and study participant details

This study does not use experimental model and subject details typical in the life sciences.

### Method details

The corresponding low-energy spin-dependent effective Hamiltonian near two Dirac points for this junction in Figure 2A is written as

(Equation 1) $$H_{\eta }^{s}=\hbar v_{F}\left(\eta \tau_{x}k_{x}+\tau_{y}k_{y}\right)+\eta \lambda_{M}\left(x\right)\tau_{0}+\eta \lambda_{\Omega }\left(x\right)\tau_{z}+s\lambda_{AF}\left(x\right)\tau_{z}+U\left(x\right)\tau_{0}$$

with *λ*_*M*,Ω,*AF*_(*x*) = *λ*_*M*,Ω,*AF*_Θ(*dx* − *x*^2^) and *U*(*x*) = *U*_*l*_Θ(−*x*) + *U*_*s*_Θ(*dx* − *x*^2^) + *U*_*l*_Θ(*x* − *d*), where *d* is the length of the scattering region. In addition, *s* = ±1 represents the spin-up and spin-down modes, *v*_*F*_ = 1 × 10^6^ *m*/*s* is the Fermi velocity, and *τ*_0,*x*,*y*,*z*_ are the sublattice Pauli matrices. The first term originates from pristine graphene with *η* = ±1 for the valleys *K* and *K*′. The second term denotes the modified Haldane model^45^ with the strength *λ*_*M*_, which is supported by various experiments.^46^^,^^47^^,^^48^ Furthermore, the modified Haldane model in real space is expressed as $t_{2}\sum_{\ll i,j\gg }e^{-i\nu_{ij}\phi }c_{i}^{†}c_{j}$,where *t*_2_ denotes the next-nearest-neighbor hopping, *ϕ* is the phase factor, and *ν*_*ij*_ = +1(−1) denotes the counterclockwise (clockwise) hopping between the sublattice A, while *ν*_*ij*_ = −1(+1) for the sublattice B. As the phase is set to *ϕ* = ±*π*/2, this term can be rewritten as $i\lambda_{M}/3\sqrt{3}\sum_{\ll i,j\gg }\nu_{ij}c_{i}^{†}c_{j}$, and its strength *λ*_*M*_ can take positive or negative values accordingly. Therefore, the corresponding low-energy effective Hamiltonian near the two Dirac points can be written as *ηλ*_*M*_*τ*_0_. The third term is the off-resonant CPL^49^ with the intensity $\lambda_{\Omega }=\mp \frac{9t^{2}{J_{1}}^{2}\left(z\right)}{\hbar \omega }$, where the positive (negative) value of *λ*_Ω_ denotes the right- (left-) polarized direction, $z=\frac{Aea}{\hbar }$ is the dimensionless light intensity, and *J*_1_ is the first-order Bessel function. According to the Floquet theory, the third term can be realized when the condition ℏ*ω*≫6*t*^50^^,^^51^ is satisfied. Moreover, when the lowest frequency ℏ*ω* = 6*t* and *z* = 0.45 are accepted, the intensity $\lambda_{\Omega }=\mp \frac{9t^{2}{J_{1}}^{2}\left(z\right)}{\hbar \omega }$ could reach about 0.2*eV*, which satisfies our accepted values of *ηλ*_Ω_*τ*_*z*_ adopted in our calculated results. The fourth term describes the AFM exchange field with the strength *λ*_*AF*_, which induces the opposite exchange potentials for the sublattices A and B. By the ferromagnetic substrates^52^^,^^53^ in graphene, this staggered exchange potential can be induced.

The eigenvalues of the Hamiltonian in Equation 1 can be generally expressed as

(Equation 2) $$E=\eta \lambda_{M}\left(x\right)+U\left(x\right)\pm \sqrt{\hbar^{2}{v_{F}}^{2}k^{2}+{\left(\eta \lambda_{\Omega }+s\lambda_{AF}\right)}^{2}}$$

where ± denotes the conduction and valence bands, respectively. The eigenvalues for the two leads and scattering region can be directly calculated by Equation 2. Furthermore, due to the translational invariance in the y direction, the general form of wave functions in three regions in Figure 2A can be expressed as

(Equation 3a) $$\varphi_{I}=\left(\begin{array}{l} \hbar v_{F}\left(\eta k_{x}^{I}-ik_{y}\right)\\ E-U_{l}\; \end{array}\right)e^{ik_{x}^{I}x+ik_{y}y}+r_{I}\left(\begin{array}{l} \hbar v_{F}\left(-\eta k_{x}^{I}-ik_{y}\right)\\ E-U_{l}\; \end{array}\right)e^{-ik_{x}^{I}x+ik_{y}y}$$

(Equation 3b) $$\varphi_{II}=t_{II}\left(\begin{array}{l} \hbar v_{F}\left(\eta k_{x}^{II}-ik_{y}\right)\\ E-\eta \lambda_{M}-\eta \lambda_{\Omega }-U_{s}-s\lambda_{AF}\; \end{array}\right)e^{ik_{x}^{II}x+ik_{y}y}+r_{II}\left(\begin{array}{l} \hbar v_{F}\left(-\eta k_{x}^{II}-ik_{y}\right)\\ E-\eta \lambda_{M}-\eta \lambda_{\Omega }-U_{s}-s\lambda_{AF}\; \end{array}\right)e^{-ik_{x}^{II}x+ik_{y}y}$$

(Equation 3c) $$\varphi_{III}=t_{III}\left(\begin{array}{l} \hbar v_{F}\left(\eta k_{x}^{III}-ik_{y}\right)\\ E-U_{l}\; \end{array}\right)e^{ik_{x}^{III}x+ik_{y}y}$$

where $k_{x}^{I},\;k_{x}^{II}$, and $k_{x}^{III}$ are the wave vectors in the left lead, scattering region, and right lead, respectively. And the parallel and perpendicular wave vectors in Equations 3a, 3b, and 3c can be further written as

(Equation 4a) $$k_{y}=\frac{\left|E-U_{l}\right|}{\hbar v_{F}}\sin\;\theta$$

(Equation 4b) $${\left(k_{x}^{I}\right)}^{2}=\frac{{\left(E-U_{l}\right)}^{2}}{{\left(\hbar v_{F}\right)}^{2}}-{k_{y}}^{2}$$

(Equation 4c) $${\left(k_{x}^{II}\right)}^{2}=\frac{{\left(E-\eta \lambda_{M}-U_{s}\right)}^{2}-{\left(\eta \lambda_{\Omega }+s\lambda_{AF}\right)}^{2}}{{\left(\hbar v_{F}\right)}^{2}}-{k_{y}}^{2}$$

(Equation 4d) $${\left(k_{x}^{III}\right)}^{2}=\frac{{\left(E-U_{l}\right)}^{2}}{{\left(\hbar v_{F}\right)}^{2}}-{k_{y}}^{2}$$

where *θ* is the incident angle from the left lead.

By using the continuity condition of the wave function at the interfaces between the leads and the scattering region, such as *ψ*_*I*_(0) = *ψ*_*II*_(0) and *ψ*_*II*_(*d*) = *ψ*_*III*_(*d*), Equations 3a, 3b, and 3c can be further transformed into a matrix form as follows

(Equation 5) AX=B,

where

(Equation 6a) $$X={\left[r_{I},t_{II},r_{II},t_{III}\right]}^{T}$$

(Equation 6b) $$B={\left[-\hbar v_{F}{\left(\eta k_{x}^{I}-ik_{y}\right)}_{I},-\left(E-U_{l}\right),0,0\right]}^{T}$$

Additionally, the matrix ***A*** can be expressed as $A={\left[\begin{array}{cccc} a_{1} & a_{2} & a_{3} & a_{4} \end{array}\right]}^{T}$, where each row of the matrix ***A*** is written as

(Equation 7a) $$a_{1}=\left[\hbar v_{F}\left(-\eta k_{x}^{I}-ik_{y}\right),-\hbar v_{F}\left(\eta k_{x}^{II}-ik_{y}\right),-\hbar v_{F}\left(-\eta k_{x}^{II}-ik_{y}\right),0\right]$$

(Equation 7b) $$a_{2}=\left[E-U_{l},-\left(E-\Delta \right),-\left(E-\Delta \right),0\right]$$

(Equation 7c) $$a_{3}=\left[0,\hbar v_{F}\left(\eta k_{x}^{II}-ik_{y}\right)e^{ik_{x}^{II}L},\hbar v_{F}\left(-\eta k_{x}^{II}-ik_{y}\right)e^{-ik_{x}^{II}L},-\hbar v_{F}\left(\eta k_{x}^{III}-ik_{y}\right)e^{ik_{x}^{III}L}\right]$$

(Equation 7d) $$a_{4}=\left[0,\left(E-\Delta \right)e^{ik_{x}^{II}L},\left(E-\Delta \right)e^{-ik_{x}^{II}L},-\left(E-U_{l}\right)e^{ik_{x}^{III}L}\right]$$

with $\Delta =\eta \lambda_{M}+\eta \lambda_{\Omega }+U_{s}+s\lambda_{AF}$. Based on Cramer’s Rule, the spin- and valley-resolved transmission coefficient can be calculated by

(Equation 8) $$t_{III}=\det\left(A_{4}\right)/\det\left(A\right)$$

where the matrix ***A***_4_ can be realized by replacing the fourth column of the matrix ***A*** with the matrix B in Equation 6b. Then, the corresponding transmission probability is described as $T_{\eta s}={\left|t_{III}\right|}^{2}$, which is a function of the incident angle *θ*. At zero temperature, the spin- and valley-resolved conductance over all possible incident angles is expressed as^38^^,^^54^

(Equation 9) $$G_{\eta s}=G_{0}\int_{-\pi /2}^{\pi /2}T_{\eta s}\left(\theta \right)\cos\;\theta d\theta$$

where $G_{0}=e^{2}Wk_{F}/2\pi h$ is the reduced unit of the conductance with the system transverse width *W* and the Fermi wave vector $k_{F}=\left|E_{F}-U_{l}\right|/\hbar v_{F}$ of the left (incident) lead. It is worth noting that with the fixed Fermi energy *E*_*F*_, *k*_*F*_ can be tuned by the electrostatic potential *U*_*l*_. Therefore, the value of *G*_0_ can be accordingly modulated by *U*_*l*_, which is discussed in the following results. Furthermore, the valley (spin) polarization is defined as

(Equation 10) $$P_{V\left(s\right)}=\left[G_{K\left(↑\right)}-G_{K^{\prime }\left(↓\right)}\right]/G_{t}$$

where $G_{t}=G_{K\left(↑\right)}+G_{K^{\prime }\left(↓\right)}$ is the total conductance. All calculated results are obtained in MATLAB R2022a.

### Quantification and statistical analysis

This study does not include quantification and statistical analysis.
